# Supplementary material for: Programmable Physical Properties of Freestanding Chitosan Membranes Electrofabricated in Microfluidics
Source: Membranes (Basel). 2023 Feb 28;13(3):294. doi: 10.3390/membranes13030294 (PMC10052736; doi:10.3390/membranes13030294)
Supplement: Supplementary file 1 [file membranes-13-00294-s001.zip › membranes-2195642-supplementary.pdf]

*membranes*

MDPI

*Supplementary Materials*

# Programmable Physical Properties of Freestanding Chitosan Membranes Electrofabricated in Microfluidics

**Khanh L. Ly [1], Piao Hu [2], Christopher B. Raub [1] and Xiaolong Luo [2,*]**

[1] Department of Biomedical Engineering, School of Engineering, Catholic University of America, Washington, DC 20064, USA
[2] Department of Mechanical Engineering, School of Engineering, Catholic University of America, Washington, DC 20064, USA
* Correspondence: luox@cua.edu

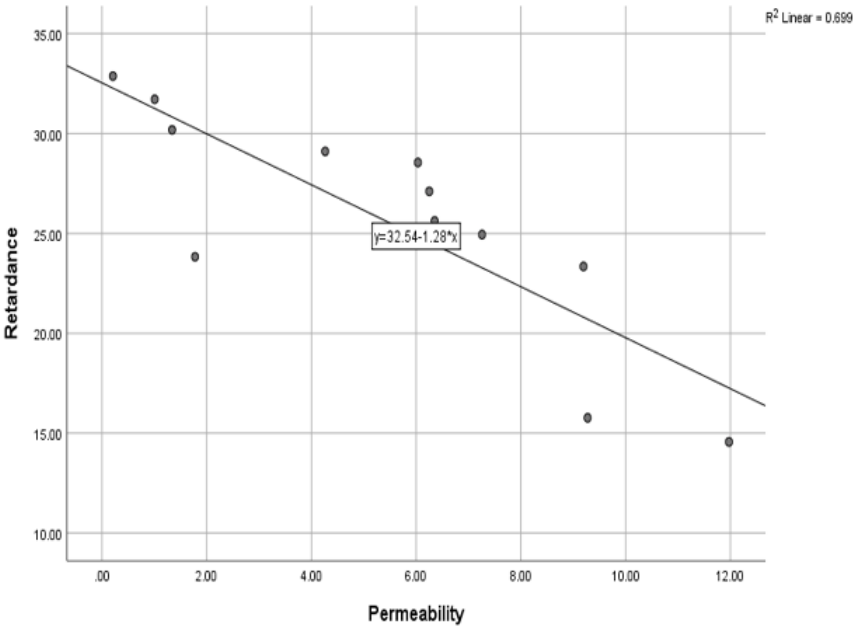


**Correlations**

| | | Permeability | Retardance |
|---|---|---|---|
| Permeability | Pearson Correlation | 1 | -.836** |
| | Sig. (2-tailed) | | .001 |
| | N | 12 | 12 |
| Retardance | Pearson Correlation | -.836** | 1 |
| | Sig. (2-tailed) | .001 | |
| | N | 12 | 12 |

**. Correlation is significant at the 0.01 level (2-tailed).

**Figure S1.** Correlation plot and test between the semi-permeability and optical retardance of electrofabricated chitosan membranes.
